# Large-Conductance Calcium-Activated Potassium Channels and Voltage-Dependent Sodium Channels in Human Cementoblasts

**DOI:** 10.3389/fphys.2021.634846

**Published:** 2021-04-20

**Authors:** Satomi Kamata, Maki Kimura, Sadao Ohyama, Shuichiro Yamashita, Yoshiyuki Shibukawa

**Affiliations:** ^1^Department of Removable Partial Prosthodontics, Tokyo Dental College, Tokyo, Japan; ^2^Department of Physiology, Tokyo Dental College, Tokyo, Japan

**Keywords:** cementoblasts, human, K_Ca_ channels, large-conductance Ca^2+^-activated K^+^ channels, voltage-dependent Na^+^ channels

## Abstract

Cementum, which is excreted by cementoblasts, provides an attachment site for collagen fibers that connect to the alveolar bone and fix the teeth into the alveolar sockets. Transmembrane ionic signaling, associated with ionic transporters, regulate various physiological processes in a wide variety of cells. However, the properties of the signals generated by plasma membrane ionic channels in cementoblasts have not yet been described in detail. We investigated the biophysical and pharmacological properties of ion channels expressed in human cementoblast (HCEM) cell lines by measuring ionic currents using conventional whole-cell patch-clamp recording. The application of depolarizing voltage steps in 10 mV increments from a holding potential (Vh) of −70 mV evoked outwardly rectifying currents at positive potentials. When intracellular K^+^ was substituted with an equimolar concentration of Cs^+^, the outward currents almost disappeared. Using tail current analysis, the contributions of both K^+^ and background Na^+^ permeabilities were estimated for the outward currents. Extracellular application of tetraethylammonium chloride (TEA) and iberiotoxin (IbTX) reduced the densities of the outward currents significantly and reversibly, whereas apamin and TRAM-34 had no effect. When the Vh was changed to −100 mV, we observed voltage-dependent inward currents in 30% of the recorded cells. These results suggest that HCEM express TEA- and IbTX-sensitive large-conductance Ca^2+^-activated K^+^ channels and voltage-dependent Na^+^ channels.

## Introduction

Cementum is a calcified tissue that is formed and deposited in layers on the surface of the tooth root by cementoblast cells derived from the dental follicle. Cementoblasts are microstructurally similar to osteoblasts, with a diameter of approximately 10 μM. They secrete both collagenous and non-collagenous matrix proteins such as fibronectin, osteopontin, and bone sialoprotein, and have an important role in hydroxyapatite mineral deposition. Cementum is part of the periodontal tissue, together with the gingiva; periodontal ligament; and alveolar bone, which holds the teeth to the jawbone (Antonio, 2013). The periodontal ligaments connect the alveolar bone and cementum through collagen fibers, which penetrate into both the cementum and bone, and are known as Sharpey’s fibers. Thus, the cementum provides a method for the attachment and binding of the collagen fibers that fix the tooth in place within the alveolar bone (Antonio, 2013).

Transmembrane signal transduction, associated with ion movement through the cell membrane, regulates various physiological and pharmacological processes in cells. For example, voltage-dependent Na^+^ channels play an important role in the generation of action potentials (Catterall et al., 2005) in excitable cells and also constitute a molecular substrate for the regulation of various cellular functions in non-excitable cells (Catterall et al., 2005; Mechaly et al., 2005), such as the mineralization of odontoblasts (Ichikawa et al., 2012) and osteoblasts (Chesnoy-Marchais and Fritsch, 1988). Voltage-dependent Na^+^ channels, shown as voltage-dependent inward currents in whole-cell recordings using the patch-clamp technique, are complexes of a pore-forming α subunit (encoding ion selectivity, conductance, and voltage sensing) that contain an α subunit family of voltage-dependent Na^+^ channels (Nav) (Goldin et al., 2000; Catterall et al., 2005). Auxiliary β-subunits of Nav modify channel gating kinetics and their voltage dependencies (Catterall et al., 2005; Wada, 2006).

Significant outward currents are carried through K^+^ channels, which are ubiquitously expressed in both excitable and non-excitable cells, and have roles in a number of diverse physiological and pathological functions (Gutman et al., 2005; Wei et al., 2005). Among these channels, Ca^2+^-activated K^+^ channels (K_Ca_) are unique in that they are gated in response to increases in concentrations of intracellular Ca^2+^ [(Ca^2+^)_i_] in a variety of cells. These K_Ca_ channels are classified into three subfamilies based on their biophysical and pharmacological properties, and molecular substrates: K_Ca_1.1, large-conductance or BK channels; K_Ca_3.1, intermediate-conductance or IK channels; and K_Ca_2.1 to K_Ca_2.3, small-conductance or SK channels (Wei et al., 2005). In odontoblasts and osteoblasts, which are hard tissue-forming cells similar to cementoblasts, plasma membrane signal transduction plays an important role in sensory reception (Shibukawa et al., 2015), neural communication (Moreau et al., 1996; Obata et al., 2007; Ma et al., 2013; Shibukawa et al., 2015; Nishiyama et al., 2016; Sato et al., 2018), and hard tissue formation (Ravesloot et al., 1990; Henney et al., 2009; Tsumura et al., 2010, 2012; Sato et al., 2013; Kimura et al., 2016; Kojima et al., 2017). To date, expression of the following diverse K^+^ channels has been observed in osteoblasts: voltage-gated K^+^ channels, inward-rectifier K^+^ channels, ATP-sensitive K^+^ channels, K_Ca_ channels (BK, IK, and SK channels; Henney et al., 2009), and two-pore-domain K^+^ channels (e.g., Kito et al., 2020). Odontoblasts also express voltage-gated K^+^ channel (Kv) subtypes Kv1.1, 1.2, and 1.6 (Kojima et al., 2017) and IK channels (Ichikawa et al., 2012). In addition, both osteoblasts and odontoblasts express tetrodotoxin-sensitive voltage-gated Na^+^ channels (Pangalos et al., 2011; Ichikawa et al., 2012). The voltage-dependent K^+^ channels expressed in odontoblasts are tetraethylammonium chloride (TEA) (non-selective K^+^ channel blocker) sensitive and are involved in dentin mineralization (Kojima et al., 2017). On the other hand, the inhibition of K_Ca_ (BK) channel activity in osteoblasts has been reported to promote bone formation (Henney et al., 2009). These findings indicate that the activities of plasma membrane ion channels regulate hard tissue formation. Although substantial induction of newly formed cementum occurs during regeneration of the periodontal ligament attaching the alveolar bone to the cementum, and during apical closure of root canals during endodontic treatment, the functional characteristics and expression of ion channels in human cementoblasts (HCEM) have not yet been described in detail.

Thus, the aim of this study was to clarify the functional expression of ionic channels, based on the pharmacological and biophysical profiles of the HCEM and their underlying cellular functions.

## Materials and Methods

### Cell Culture

An immortalized cell line of HCEM was used. The HCEM were provided by Professor Takashi Takata (Hiroshima University Graduate School of Dentistry); characteristics of these cementoblasts have been reported previously in Kitagawa et al. (2006). Cells were maintained in alpha-minimum essential medium, containing 10% fetal bovine serum, 1% penicillin/streptomycin (Life Technologies, Tokyo, Japan), and amphotericin B (Sigma Aldrich, St. Louis, MO, United States) at 37 °C in a humidified atmosphere of 5% CO_2_.

### Whole-Cell Patch-Clamp Recordings

Whole-cell recordings of the HCEM were performed in voltage-clamp mode using patch-clamp recordings following conventional methods (Hamill et al., 1981). Patch pipettes with a resistance of 3–8 MΩ were made from glass capillaries (DMZ-Universal Puller, Zeitz-Instruments, Martinsried, Germany) and filled with an intracellular solution (ICS). When the patch pipette was attached to the plasma membrane, the seal resistance between the pipette and membrane was measured. The average initial seal resistance was 7.4 ± 1.2 GΩ (the “giga ohm seal”; *N* = 61). The values of cell membrane resistance during whole-cell recordings were calculated from the current amplitude, induced using a depolarizing voltage step of 10 mV from a holding potential (Vh) of 0 mV. All currents were measured with an amplifier for patch clamp recording (L/M-EPC-7 plus; HEKA Elektronik, Lambrecht, Germany). After digitizing the analog current signal at 3 kHz (Digidata 1440A; Molecular Devices, Sunnyvale, CA, United States), the current traces were monitored and stored using pCLAMP software (Molecular Devices, Sunnyvale, CA, United States). Data were analyzed using pCLAMP and the technical graphics/analysis program ORIGIN (OriginLab Corporation, Northampton, MA, United States). All experiments were performed at room temperature (27 (°C). The membrane capacitance of the HCEM was calculated using a capacitive transient, induced with a depolarization step (10 10 mV) starting from a Vh of 0 0 mV. Small differences in the sizes of the HCEM were accounted for normalizing the current using measured capacitance. We expressed current amplitude in terms of current density (pA/pF).

### Solutions and Reagents

Solution compositions used in this study were shown in Table 1. A Krebs solution containing 136 mM NaCl, 5 mM KCl, 2.5 mM CaCl_2_, 0.5 mM MgCl_2_, 10 mM HEPES, 10 mM glucose, and 12 mM NaHCO_3_ (pH 7.4/Tris) was used as the standard extracellular solution (ECS). The ICS was composed of 140 mM KCl, 10 mM NaCl, and 10 mM HEPES (pH 7.2/Tris). To eliminate Cl^–^ conductance from the whole-cell currents, we prepared ECS/ICS in which NaCl and KCl were substituted with equimolar concentrations of Na-gluconate and K-gluconate, respectively (gluc-ECS/ICS). To examine whether the recorded currents were carried by K^+^, we also prepared an ICS in which KCl was substituted with an equimolar concentration of CsCl (Cs-ICS). To investigate the contribution of Ca^2+^ to the activation of recorded currents, we prepared an ECS in which Ca^2+^ was removed (Ca^2+^free-ECS). To eliminate both K^+^ and Cl^–^ conductances, we prepared an ECS and ICS using an equimolar substitution of KCl and NaCl with Cs-gluconate and Na-gluconate, respectively (Cs-gluc-ECS/ICS). For pharmacological experiments, we used TEA (Wako Pure Chemicals, Osaka, Japan) as a non-specific K^+^ channel blocker, iberiotoxin (IbTX; PEPTIDE INSTITUTE, INC., Osaka, Japan) as a large-conductance Ca^2+^-activated K^+^ channel blocker, apamin (PEPTIDE INSTITUTE) as a small-conductance Ca^2+^-activated K^+^ channel blocker, and TRAM-34 (Sigma Aldrich, St. Louis, MO, United States) as an intermediate-conductance Ca^2+^-activated K^+^ channel blocker.

**TABLE 1:** Composition of extracellular and intracellular solution.

(In mM)	NaCl	Na-gluc	KCl	K-gluc	CsCl	Cs-gluc	CaC2	MgCl2	HEPES	Glucose	NaHCO3	pH
Standard-ECS	136	0	5	0	0	0	2.5	0.5	10	10	12	7.4
Gluc-ECS	0	136	0	5	0	0	2.5	0.5	10	10	12	7.4
Ca^2+^free-ECS	140	0	5	0	0	0	0	0.5	10	10	12	7.4
Cs-Gluc-ECS	0	136	0	0	0	5	2.5	0.5	10	10	12	7.4
Standard-ECS with 5 mM [K^+^]_o_	136	0	5	0	0	0	2.5	0.5	10	10	12	7.4
Standard-ECS with 10 mM [K^+^]_o_	131	0	10	0	0	0	2.5	0.5	10	10	12	7.4
Standard-ECS with 50 mM [K^+^]_o_	91	0	50	0	0	0	2.5	0.5	10	10	12	7.4
Standard-ECS with 100 mM [K^+^]_o_	41	0	100	0	0	0	2.5	0.5	10	10	12	7.4
Gluc-ECS with 5 mM [K^+^]_o_	0	136	0	5	0	0	2.5	0.5	10	10	12	7.4
Gluc-ECS with 10 mM [K^+^]_o_	0	131	0	10	0	0	2.5	0.5	10	10	12	7.4
Gluc-ECS with 50 mM [K^+^]_o_	0	91	0	50	0	0	2.5	0.5	10	10	12	7.4
Gluc-ECS with 100 mM [K^+^]_o_	0	41	0	100	0	0	2.5	0.5	10	10	12	7.4
Standard-ICS	10	0	140	0	0	0	0	0	10	0	0	7.2
Gluc-ICS	0	10	0	140	0	0	0	0	10	0	0	7.2
Cs-ICS	10	0	0	0	140	0	0	0	10	0	0	7.2
Cs-Gluc-ICS	0	10	0	0	0	140	0	0	10	0	0	7.2

*gluc, gluconate; ECS, extracellular solution; ICS, intracellular solution.*

### Statistical Analysis

Results are expressed as the mean ± standard error (SE) of the number of tested cells (N) for which measurements were taken. The Wilcoxon signed-rank test, Friedman test, and Dunn’s *post hoc* test were used to evaluate non-parametric statistical significance. *P*-values of <0.05, were considered significant (GraphPad Prism T.O, GraphPad software, La Jolla, CA, United States).

## Results

### Passive Plasma Membrane Properties of Human Cementoblasts

We applied patch-clamp recordings to HCEM cells. The resting membrane potential of the cementoblasts in the standard-ECS/ICS, was −51.5 ± 1.3 mV (*N* = 19), which showed a relative shift toward a positive potential compared to the K^+^ equilibrium potential for this condition. The membrane capacitance was 6.6 ± 0.5 pF (*N* = 51) for the standard-ECS/ICS.

### Outward Currents in Human Cementoblasts Under Standard-ECS/ICS Conditions

**FIGURE 1:**
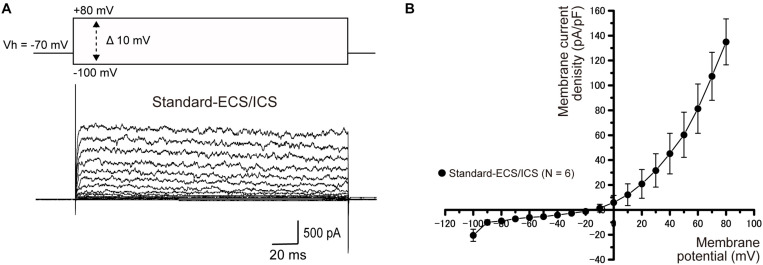
Outward currents under the standard-ECS/ICS. **(A)** Using the standard-ECS/ICS, the application of pulses of voltage in steps that ranged from –100 mV to +80 mV at 10 mV increments, with a holding potential (Vh) of –70 mV (400 ms in duration; upper traces) evoked outward currents (lower traces). **(B)** Current-voltage (I-V) relationships of the outward currents are shown. The horizontal axis indicates the membrane potential applied and the vertical axis represents the current density. I-V curves were obtained by plotting the values of the peak current amplitude as densities against the applied membrane potentials. Each point represents the mean ± SE current density of six cells.

Applying (400 ms in duration) pulses of voltage in steps that ranged from −100 to +80 mV, at 10 mV increments with a holding potential (Vh) of −70 mV evoked large outward currents in the cementoblasts maintained in standard-ECS/ICS (Figure 1A). The current–voltage (I–V) relationship demonstrated outward rectification at positive membrane potentials, with small inward currents at negative membrane potentials. The outward-rectifying currents were activated at approximately −10 mV. At a membrane potential of +80 mV, the peak current densities of the outward current were 135 ± 18.4 pA/pF (Figure 1B; *N* = 6).

### Intracellular Cs^+^ Abolishes Outward Currents

**FIGURE 2:**
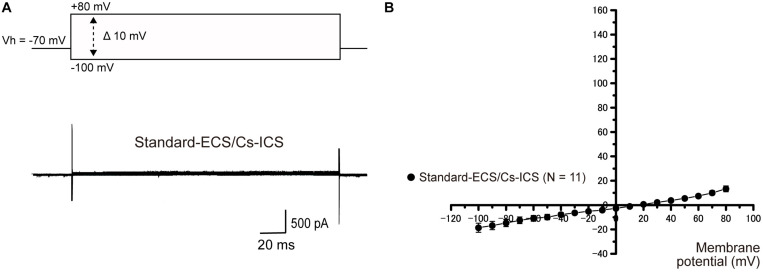
Effect of Cs^+^ on outward currents**. (A)** In the standard-ECS and Cs-ICS, outward currents almost disappeared (lower traces), compared to them recorded with standard-ICS (Figure 1). The currents were elicited by applying voltage in 10 mV increments from –100 mV to +80 mV at a holding potential (Vh) of –70 mV (400 ms in duration). **(B)** Current-voltage (I-V) relationships of the currents recorded from the Cs-ICS and standard-ICS. Each point represents the mean ± SE current densities of 11 cells.

Intracellular Cs^+^ does not permeate the pore region of K^+^ channels. To examine whether the currents recorded from HCEM were carried by K^+^, intracellular K^+^ was substituted with an equimolar concentration of Cs^+^ in the standard-ICS (Cs-ICS). When applied Cs-ICS to the cementoblasts with standard-ECS, instead of the standard-ICS, the outward currents [when pulses of voltage were applied in steps that ranged from −100 mV to +80 mV, at 10 mV increments with a holding potential (Vh) of −70 mV] almost disappeared (Figures 2A,B; *N* = 11), but showed a small residual current component. The peak current densities of the outward current at a membrane potential of +80 mV were 13.1 ± 2.5 pA/pF (*N* = 11).

### Ion Selectivity of the Currents in Human Cementoblasts

**FIGURE 3:**
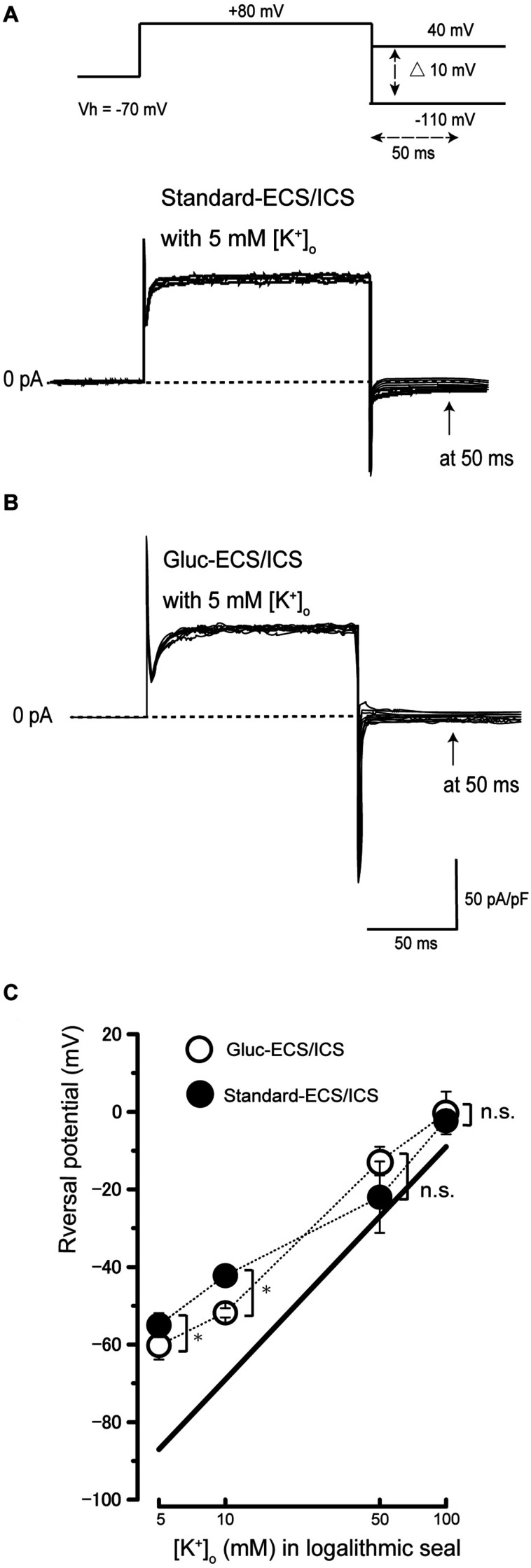
Tail current analysis of outward currents in human cementoblasts. **(A,B)** Representative current traces recorded from standard-ECS/ICS [lower traces in panel **(A)**] and gluc-ECS/ICS (B) are shown. Currents were elicited by applying voltage in steps from a Vh of –70 mV to +80 mV before hyperpolarizing in 10 mV increments ranging from –110 to +40 mV [upper traces in panel **(A)**]. To analyze the values of reversal potential, we measured the amplitudes at 50 ms [arrows in panels **(A,B)**] after initiating hyperpolarized voltage pulsing [dashed double headed arrow in upper in panel **(A)**] under both conditions. **(C)** Semilogarithmic plots of reversal potential against various values of [K^+^]_o_ (5–100 mM) under standard-ECS/ICS (filled circles) and gluc-ECS/ICS (open circles) conditions. The reversal potentials under both conditions deviated from the expected values, which were estimated for pure K^+^ conductance using the Nernst equation (solid line). Each point represents the mean ± SE reversal potential of six cells.

To examine the ionic selectivity of the recorded currents, we conducted a tail current analysis. Representative tail current traces with 5 mM [K^+^]_o_ recorded from the cementoblasts maintained in standard-ECS and standard-ICS (Figure 3A), or gluc-ECS and gluc-ICS (Figure 3B) were elicited by applying pulses of voltage in steps that ranged from a Vh of −70 mV to +80 mV. Subsequently, hyperpolarizing voltage was applied in steps that ranged from −110 to +40 mV in 10 mV increments (top traces in Figure 3A). We then measured the amplitudes of the current densities at 50 ms (arrows in both Figures 3A,B). We also measured the reversal potentials by plotting the I-V relationships of the tail currents with 5, 10, 50, and 100 mM extracellular K^+^ concentrations [(K^+^)_o_] (not shown). The mean reversal potential values based on the standard-ECS and standard-ICS were −55.0 ± 3.1 mV in 5 mM [K^+^]_o_, −42.2 ± 1.7 mV in 10 mM [K^+^]_o_, −22.0 ± 9.2 mV in 50 mM [K^+^]_o_, and −2.0 ± 2.5 mV in 100 mM [K^+^]_o_ (Figure 3C). The values based on the gluc-ECS/ICS (i.e., with extracellular and intracellular Cl^–^ eliminated from both standard environments) were −60.2 ± 3.7 mV in 5 mM [K^+^]_o_, −51.8 ± 1.2 mV in 10 mM [K^+^]_o_, −12.7 ± 3.7 mV in 50 mM [K^+^]_o_, and −0.3 ± 5.5 mV in 100 mM [K^+^]_o_ (Figure 3C). Semi logarithmic plots of the reversal potentials against [K^+^]_o_ (5–100 mM) revealed that the mean reversal potential values under both conditions deviated from those expected for a current with pure K^+^ conductance, as estimated by the Nernst equation at 27 °C, assuming an intracellular K^+^ concentration [(K^+^)_i_] of 140 mM and various [K^+^]_o_. In addition, we did not observe any significant differences in reversal potentials between the conditions (i.e., standard-ECS/ICS vs. gluc-ECS/ICS) when [K^+^]_o_ were at 50 and 100 mM. However, significant differences were observed at 5 and 10 mM [K^+^]_o_. These results suggest that Cl^–^ permeability does not seem to contribute to the outward current recorded from HCEM. Thus, we considered the Na^+^ permeability, and estimated both K^+^ and Na^+^ permeability (but not Cl^–^ permeability) using the Goldman-Hodgkin-Katz equation when the reversal potentials recorded from the cementoblasts maintained in gluc-ECS and gluc-ICS. When [Na^+^]_i_ was set to 10 mM and the [K^+^]_i_ was 140 mM, Na^+^ permeability was calculated for four different [K^+^]_o_ and extracellular Na^+^ concentrations [(Na^+^)_o_]. The estimated Na^+^ permeabilities (P_Na_) were: 0.06 in 5 mM [K^+^]_o_ and 136 mM [Na^+^]_o_; 0.07 in 10 mM [K^+^]_o_ and 131 mM [Na^+^]_o_; 0.40, in 50 mM [K^+^]_o_ and 91 mM [Na^+^]_o_; and 0.8, in 100 mM [K^+^]_o_ and 41 mM [Na^+^]_o_; and K^+^ permeability (P_k_) was set to 1.0. For the standard-ECS/ICS, the estimated P_Na_ were 0.09, in 5 mM [K^+^]_o_ and 136 mM [Na^+^]_o_; 0.14 in 10 mM [K^+^]_o_ and 131 mM [Na^+^]_o_; 0.1, in 50 mM [K^+^]_o_ and 91 mM [Na^+^]_o_; and 0.95, in 100 mM [K^+^]_o_ and 41 mM [Na^+^]_o_; and K^+^ permeability (P_k_) was set to 1.0. There were no significant differences in the P_Na_ values of the cementoblasts in the standard-ECS/ICS and gluc-ECS/ICS. These results suggest that the currents recorded from HCEM were carried by K^+^ with background Na^+^ conductances.

### Outward Currents Are Sensitive to Extracellular Ca^2+^

**FIGURE 4:**
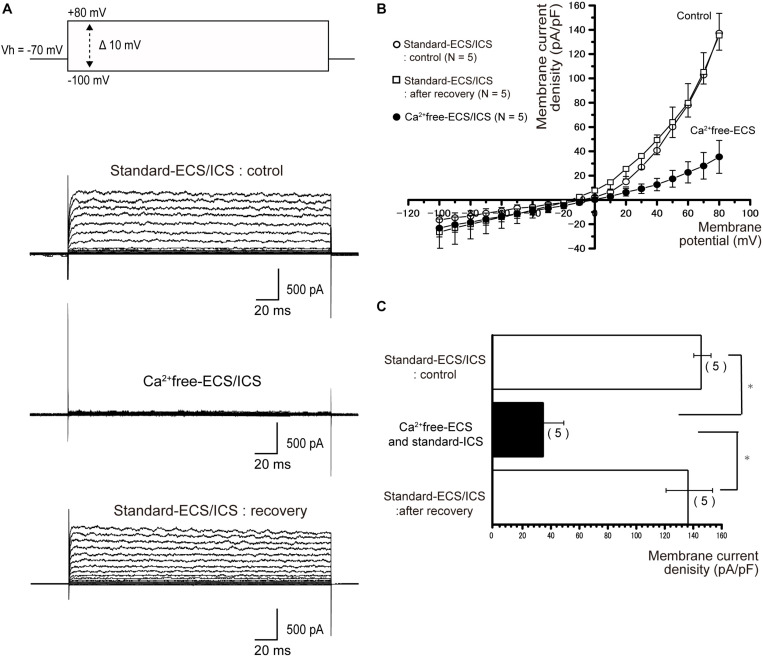
Outward currents were extracellular Ca^2+^ sensitive. **(A)** To examine Ca^2+^-dependent processes in the activation of outward currents, extracellular Ca^2+^ was eliminated (Ca^2+^free-ECS). The currents were elicited by applying (400 ms) voltage in 10 mV increments from –100 mV to +80 mV at a holding potential (Vh) of –70 mV. The second upper traces show the currents recorded from the standard-ECS/ICS, the second lower traces show those based on the Ca^2+^free-ECS/standard-ECS, and the lower traces represent those after extracellular Ca^2+^ was restored. **(B)** Current-voltage (I-V) relationships for the currents recorded with the standard-ECS/ICS (open circles), Ca^2+^free-ECS and standard-ICS (filled circles), and after recovery. Points represent the mean ± SE current density of five cells. When we subtracted the current amplitudes in the Ca^2+^free-ECS from those obtained in the standard-ECS, the conductance of the pure Ca^2+^-activated currents was 293 pS. **(C)** Summary bar graph showing the current densities at a membrane potential of +80 mV, under the conditions of the standard-ECS/ICS (upper column), Ca^2+^free-ECS and standard-ICS, and standard-ECS/ICS as recovery. Statistically significant differences are indicated by asterisks, ^∗^*P* < 0.05.

To investigate the Ca^2+^-activated processes in current generation, we first recorded the outward currents of the cementoblasts in the standard-ECS/ICS (Figure 4A), then recorded the currents based on the solution in which extracellular Ca^2+^ had been removed (Ca^2+^free-ECS; Figure 4A). The currents were almost completely and reversibly abolished in the cementoblasts in the Ca^2+^ free-ECS (Figures 4A,B). The current densities were measured at a membrane potential of +80 mV; the values recorded using the solution in which extracellular Ca^2+^ had been removed (35.5 ± 13.5 pA/pF; *N* = 5) were significantly lower than those recorded for the standard-ECS (137.2.4 ± 16.3 pA/pF; *N* = 5). When extracellular Ca^2+^ was re-administrated into the ECS, the current density was completely restored (135.3 ± 12.1 pA/pF; *N* = 5) to the same level as recorded for the standard-ECS/ICS (Figure 4C; *N* = 5). To obtain pure Ca^2+^-activated current conductance, we subtracted the current amplitudes in the Ca^2+^free-ECS from those obtained in the standard-ECS. The conductance of the pure Ca^2+^-activated currents was 228 ± 29.2 pS (*N* = 5).

### Outward Currents Are Sensitive to Extracellular Non-specific and Specific Antagonists for Large-Conductance Ca^2+^-Activated K^+^ Channels

**FIGURE 5:**
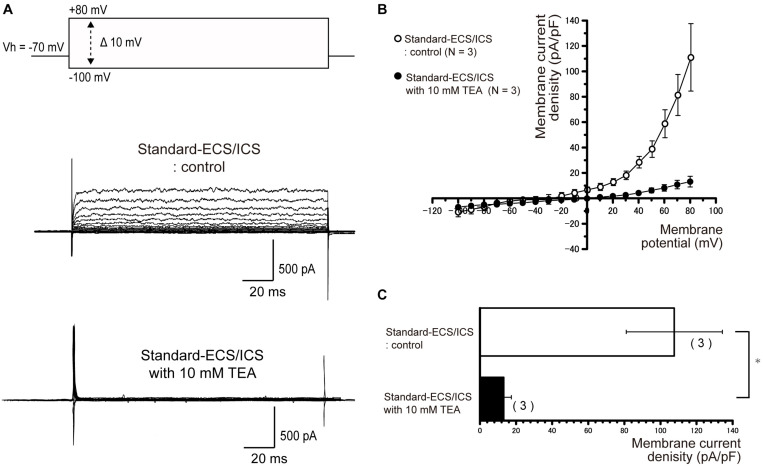
Outward rectifying currents in human cementoblasts were TEA sensitive**. (A)** Representative current traces recorded under standard-ECS/ICS conditions, both without (second upper traces) and with the application of 10 mM TEA (third upper traces) are shown. Currents were evoked by applying (400 ms) voltage in 10 mV increments from –100 mV to +80 mV at a holding potential (Vh) of –70 mV (upper traces). **(B)** Current-voltage (I-V) relationships for the currents recorded under standard-ECS/ICS conditions without (open circles) and with the application of 10 mM TEA (filled circles) are shown. Each point represents the mean ± SE current density of three cells. **(C)** Summary bar graphs showing current densities under standard-ECS/ICS conditions at a membrane potential of +80 mV without (upper column) and with the application of 10 mM TEA (lower column). Columns represent the mean ± SE of three cells. Statistically significant differences are denoted by asterisks, ^∗^*P* < 0.05.

**FIGURE 6:**
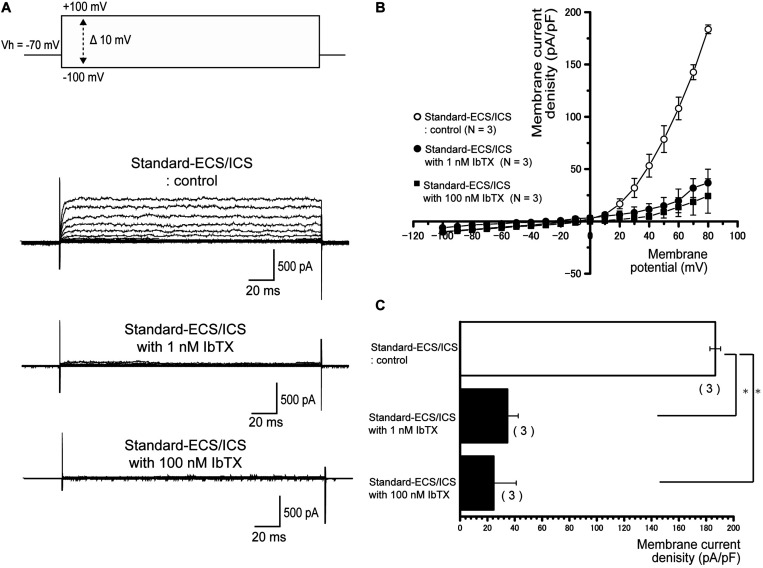
Outward currents are sensitive to large-conductance Ca^2+^-activated K^+^ channel antagonists**. (A)** Representative current traces recorded under standard-ECS/ICS conditions without (second upper traces) and with the application of 1 and 100 nM IbTX (third and fourth upper traces, respectively) are shown. Currents were evoked by applying (400 ms) voltage in 10 mV increments from –100 mV to +80 mV at a holding potential (Vh) of –70 mV (upper traces). **(B)** Current-voltage (I-V) relationships for the currents recorded under standard-ECS/ICS conditions without (open circles) or with the application of 1 nM IbTX (filled circles) and 100 nM IbTX (filled squares) are shown. Each point represents the mean ± SE current of three cells. **(C)** Summary bar graph showing current densities under standard-ECS/ICS conditions at a membrane potential of +80 mV without (upper column) or with the application of 1 nM IbTX (middle column) and 100 nM IbTX (lower column). Each column represents the mean ± SE of each three cells. Statistically significant differences are denoted by asterisks, ^∗^*P* < 0.05.

In the cementoblasts maintained in the standard-ECS/ICS, the application of 10 mM extracellular TEA significantly reduced the outward current amplitude (Figure 5A) at membrane potentials ranging from −20 mV to +80 mV, in comparison to that recorded from the standard-ECS/ICS without TEA (Figures 5A–C; *N* = 3). In addition, the application of a large-conductance Ca^2+^-activated K^+^ channel blocker, IbTX (1 and 100 nM), also significantly reduced the outward current densities (Figure 6A) at positive membrane potentials (Figure 6B). The current densities at the membrane potential of −80 mV following application of 100 nM IbTX (24.7 ± 16.4 pA/pF; *N* = 3) and 1 nM IbTX (37 ± 13 pA/pF; *N* = 3) were significantly lower than that measured from the control without IbTX (183.6 ± 4.2 pA/pF; *N* = 3) (Figure 6C; *N* = 3).

### Apamin and TRAM-34 Have No Effect on Outward Currents

**FIGURE 7:**
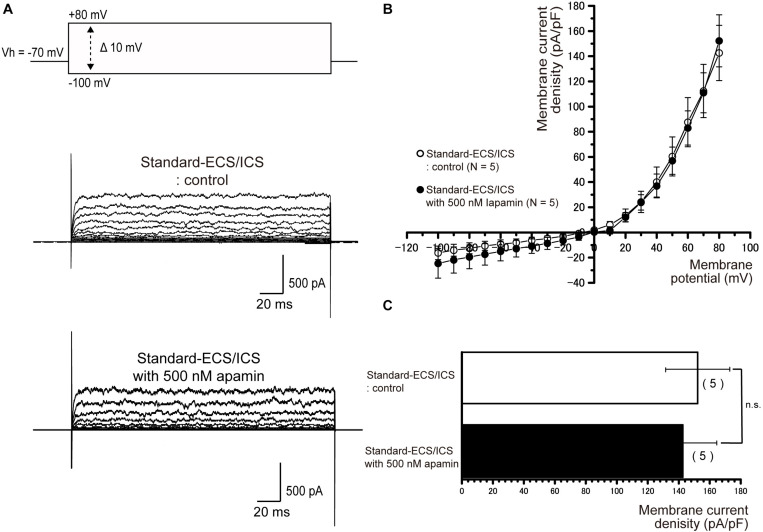
Apamin had no effects on the outward currents. **(A)** Representative current traces recorded under standard-ECS/ICS conditions without (second upper traces) and with the application of 500 nM apamin (third upper traces) are shown. Currents were evoked by applying (400 ms) voltage in 10 mV increments from –100 mV to +80 mV at a holding potential (Vh) of –70 mV (upper traces). **(B)** Current-voltage (I-V) relationships for the currents recorded under standard-ECS/ICS conditions without (open circles) and with the application of 500 nM apamin (filled circles) are shown. Each point represents the mean ± SE current density of five cells. **(C)** Summary bar graphs showing current densities under standard-ECS/ICS conditions at a membrane potential of +80 mV without (upper column) and with the application of 500 nM apamin (lower column). Each column indicates the mean ± SE of five cells.

**FIGURE 8:**
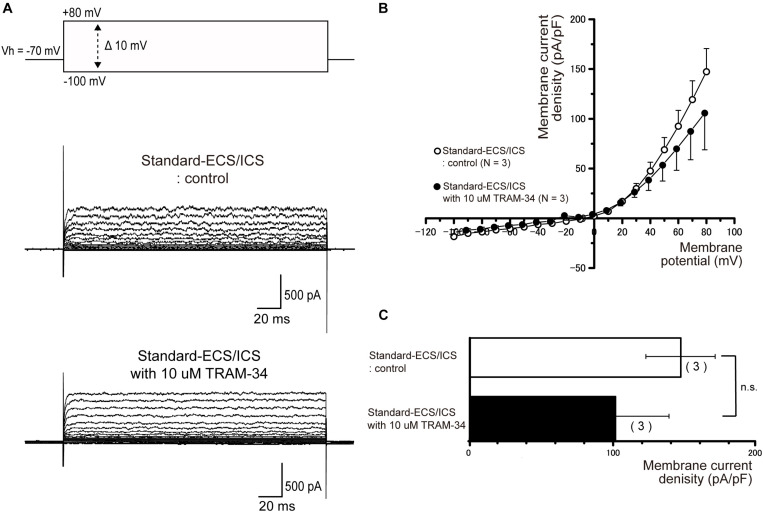
TRAM-34 had no effect on the outward currents. **(A)** Representative current traces recorded under standard-ECS/ICS conditions without (second upper traces) and with the application of 10 μM TRAM-34 (third upper traces) are shown. Currents were evoked by applying (400 ms) voltage in 10 mV increments from –100 mV to +80 mV at a holding potential (Vh) of –70 mV (upper traces). **(B)** Current-voltage (I-V) relationships for the currents recorded under standard-ECS/ICS conditions without (open circles) and with the application of 10 μM TRAM-34 (filled circles) are shown. Each point represents the mean ± SE current density of the three cells. **(C)** Summary bar graphs showing current densities under standard-ECS/ICS conditions at a membrane potential of +80 mV without (upper column) and with application of 10 μM TRAM-34 (lower column). Each column represents the mean ± SE of the three cells.

In the cementoblasts maintained in standard-ECS/ICS, application of the small-conductance Ca^2+^-activated K^+^ channel blocker, apamin (500 nM), and the intermediate-conductance Ca^2+^-activated K^+^ channel blocker, TRAM-34 (10 μM), did not have any significant effect on the outward currents elicited by the voltage protocols shown in Figures 4–6 (Figures 7, 8).

### Inward Currents Under Cs-gluc-ECS/ICS

**FIGURE 9:**
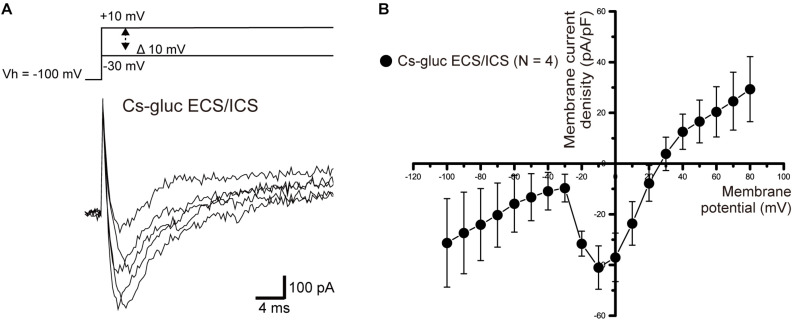
Inward currents under the Cs-Gluc-ECS/ICS**. (A)** Under the Cs-gluc-ECS/ICS, when (400 ms) voltage was applied in 10 mV increments from –30 to +10 mV at a Vh of –100 mV (upper traces), an inward current component appeared. **(B)** Current-voltage relationships (I-V curves) of the inward currents are shown. The horizontal axis indicates the membrane potential applied and the vertical axis represents the current density. I-V curves were obtained by plotting the values of the peak current amplitude as densities against the applied membrane potentials. Each point represents the mean ± SE current density of four cells.

We measured ionic currents of the cementoblasts in Cs-gluc-ECS/ICS to eliminate the contribution of K^+^ and Cl^–^ conductances. When we applied voltage steps −30 to +10 mV from a Vh of −100 mV, an inward current component was successfully observed in three out of 10 cells (Figure 9A). The inward currents were activated at a membrane potential of −30 mV and reached maximum current densities at −10 mV (Figure 9B). However, we could not record the inward currents when the Vh was set to −70 mV (data not shown), suggesting that the currents were carried by voltage-dependent Na^+^ channels.

## Discussion

In the present study, we showed large-conductance Ca^2+^-activated K^+^ currents in HCEM. When the cells are maintained under standard physiological conditions (i.e., in the standard-ECS/ICS), the outward rectifying currents are sensitive to extracellular TEA-, IbTX-, and Ca^2+^. TEA is a non-selective K^+^ channel blocker, which affects a number of K^+^ channel families, including voltage-dependent K^+^ channels as well as large- and small- [K_Ca_2.3; (Wittekindt et al., 2004)] conductance Ca^2+^-activated K^+^ channels. The recorded outward currents did not appear to be sensitive to apamin, as small-conductance Ca^2+^-activated K^+^ channel (K_Ca_2.1–K_Ca_2.3) blockers. TRAM-34, a potent and selective inhibitor of intermediate-conductance Ca^2+^-activated K^+^ channels (K_Ca_3.1) (Agarwal et al., 2013) also had no effect on the outward currents of HCEM. The half-maximal (50%) inhibitory concentrations (IC_50_) of apamin for human small-conductance Ca^2+^-activated K^+^ channel expressed cell lines are 3–8 nM for K_Ca_2.1 (Shah and Haylett, 2000; Strøbaek et al., 2000), 0.3 nM for K_Ca_2.2 (Jäger et al., 2000), and 0.8–13 nM for K_Ca_2.3 (Terstappen et al., 2001; Wittekindt et al., 2004). For TRAM-34, IC_50_ has been demonstrated for the intermediate-conductance Ca^2+^-activated K^+^ channel, which is expressed in human T lymphocytes at 20–25 nM. In the present study, higher concentrations of apamin (500 nM) and TRAM-34 (10 μM) were used than in previous studies (shown above), both of which had no significant effects on outward-rectifying currents. This indicates that HCEM do not express small- and intermediate-conductance Ca^2+^-activated K^+^ channels. IbTX is a selective blocker of the large-conductance Ca^2+^-activated K^+^ channel, K_Ca_1.1 (Candia et al., 1992), and has an IC_50_ of approximately 1 nM (Candia et al., 1992). Following the application of 100 nM IbTX, which is 100 times higher than the reported IC_50_, the observed outward current amplitudes were significantly reduced than those recorded without IbTX. These results demonstrate that outward currents in HCEM are carried by large-conductance Ca^2+^-activated K^+^ channels. In addition, the conductance of the pure Ca^2+^-activated currents was 228 pS, falling within that of large-conductance Ca^2+^-activated K^+^ channels (Wei et al., 2005).

The mean value of the resting membrane potential of the cementoblasts was approximately −51.5 mV, with a membrane capacitance of 6.6 pF. The resting membrane potential value of the cells maintained in the standard-ECS/ICS showed a positive shift from the K^+^ equilibrium potential, which was predicted by the solutions in the presence of 5 mM [K^+^]_o_ (where [K^+^]_i_ was 140 mM). In the tail current analysis, the reversal potentials of the cementoblasts maintained in the gluc-ECS/ICS (Cl^–^ in the ECS/ICS replaced with gluconate; extracellular and intracellular Cl^–^ concentrations were 6 and 0 mM, respectively) also deviated from the pure K^+^ conductance. There were no differences between the values of the reversal potentials of the standard-ECS/ICS and gluc-ECS/ICS. These results indicate that Cl^–^ conductances do not contribute to the outward currents in HCEM. Thus, we calculated Na^+^ permeability using the Goldman-Hodgkin-Katz equation from the reversal potentials with various [K^+^]_o_, for the cementoblasts in standard-ECS/ICS and gluc-ECS/ICS (K^+^ permeability was set to 1.0). The Na^+^ permeabilities ranged from 0.09 to 0.95 in the standard-ECS/ICS and 0.06–0.8 in the gluc-ECS/ICS. There were no significant differences in these P_Na_ values estimated in various [K^+^]_o_ between both ECS and ICS condition. Therefore, both K^+^ and background Na^+^ conductance likely contributed to the outward currents in cementoblasts. This suggests that background Na^+^ conductance might be involved in the depolarized shift of resting membrane potentials from the potential mediated by the pure K^+^ conductance. Further studies are required to clarify the nature of this Na^+^ conductance, such as whether it is driven by the Na^+^-Ca^2+^ exchangers that are responsible for the Ca^2+^ extrusion pathway for mineralization, as reported for odontoblasts (Tsumura et al., 2010, 2012; Sato et al., 2013).

When Cl^–^ and K^+^ were substituted with gluconate^–^ and Cs^+^, respectively, we observed voltage-dependent inward currents in 30% of cementoblasts. The half-maximal inactivation potential (V_0.5_), which describes the membrane potential where 50% of the membrane Na^+^ channels are inactivated, has been reported as below −80 mV (Catterall et al., 2005; Dib-Hajj et al., 2009). These results are in line with those from our study; inward currents were observed at Vh of −100 mV, but not at Vh of −70 mV. This also implies that cementoblasts express functional voltage-dependent Na^+^ channels (Nav) that are inactivated at the physiological resting membrane potential of cementoblasts. In other words, the Nav are activated when cells exhibit a negative “hyperpolarized” membrane potential. One possible explanation (Ichikawa et al., 2012) for this is that activation of Ca^2+^-activated K^+^ channel openings occurs to elicit hyperpolarized membrane potentials, which are capable of activating Nav in cementoblasts. However, further research is required to clarify whether cementoblasts express Nav, and the regulatory mechanisms involved in the activation of both Nav and the Ca^2+^-dependent processes involved in the activation of outward currents through Ca^2+^-activated K^+^ channels. Experimental work should be aimed at clarifying the Ca^2+^ signaling pathway, which is responsible for the activation of Ca^2+^-activated K^+^ channels. Cementoblasts express cation (such as Ca^2+^) permeable transient receptor potential ankyrin subfamily member 1 (TRPA1) channels, which are known to be sensitive to alkaline extracellular conditions (Tsumura et al., 2013; Kimura et al., 2016) to mediate proliferation and cementum mineralization (Muramatsu et al., 2019). Although confirmation is needed, cementoblast K_Ca_ channel activation may play a particularly important role in cell proliferation and differentiation, and cementum formation, *via* crosstalk with intracellular Ca^2+^ signaling pathways mediated by high pH-sensitive TRP channel subfamily members (Kimura et al., 2018). TRPA1 channels also mediate mechanosensitive Ca^2+^ signaling (Sato et al., 2013, 2018; Tsumura et al., 2013; Shibukawa et al., 2015; Kimura et al., 2016). With regard to cementoblast cellular functions, detailed mechanosensitive-Ca^2+^ signaling in cementoblasts is also of immediate interest, since cementoblasts located at the surface of the cementum are frequently exposed to mechanical stress from the tooth socket during mastication or orthodontic treatment. Additional experiments using Alizarin red and/or von Kossa staining *in vitro* are needed to clarify the contribution of large-conductance Ca^2+^-activated K^+^ channels to mineralization processes (Kimura et al., 2016; Kojima et al., 2017) with or without biophysical or pharmacological stimuli mimicking the *in vivo* environment in which cementoblasts function.

In conclusion, we have described the expression of large-conductance Ca^2+^-activated K^+^ channels in HCEM. Outward currents showed K^+^ conductance with background Na^+^ conductance. We also observed voltage-dependent inward currents, which might be carried by Na^+^, due to its electrophysiological properties. The ionic channels expressed in HCEM may play a specific role in driving cellular functions, such as cementogenesis.

## Data Availability Statement

The original contributions presented in the study are included in the article/supplementary material, further inquiries can be directed to the corresponding author/s.

## Author Contributions

SK, MK, and YS carried out the patch-clamp study. YS, SO, and SY participated in the design of the study. SK, SO, and MK performed the statistical analysis. YS and SY conceived of the study, and participated in its design and coordination, and helped to draft the manuscript. All authors read and approved the final manuscript.

## Conflict of Interest

The authors declare that the research was conducted in the absence of any commercial or financial relationships that could be construed as a potential conflict of interest.
